# Serum trough concentration threshold and risk factors of cefoperazone-induced coagulopathy in critically ill patients: A retrospective case-control study

**DOI:** 10.1007/s00228-024-03634-4

**Published:** 2024-02-14

**Authors:** Qian Wang, Pei Liang, Ying Xu, Binbin Yuan, Chen Lan, Xiaodi Yan, Li Li

**Affiliations:** 1 Department of Pharmacy, Nanjing Drum Tower Hospital, School of Basic Medicine and Clinical Pharmacy, China Pharmaceutical University, Nanjing, Jiangsu China; 2 Department of Pharmacy, Drum Tower Hospital Affiliated to Medical School of Nanjing University, Nanjing, Jiangsu China; 3 Intensive Care Unit, Drum Tower Hospital Affiliated to Nanjing University School of Medicine, Nanjing, Jiangsu China; 4 School of Basic Medicine and Clinical Pharmacy, China Pharmaceutical University, Nanjing, Jiangsu China

**Keywords:** Cefoperazone, Coagulopathy, Serum trough concentration, Risk factors

## Abstract

**Purpose:**

To analyze the risk factors influencing the development of cefoperazone-induced coagulopathy in critically ill patients and determine the threshold of serum trough concentration.

**Methods:**

A retrospective case-control study was conducted in the intensive care unit patients treated with cefoperazone, and it was approved by the Ethical Committee of Drum Tower Hospital affiliated with the Medical School of Nanjing University (NO.2023-158-01). Patients were divided into the normal group and coagulopathy group based on prothrombin time. The clinical characteristics of the two groups were compared using univariate analysis. The serum concentration threshold and influencing factors of cefoperazone-induced coagulopathy in critically ill patients were analyzed using the receiver operating characteristic curve and multivariate logistic regression analysis.

**Results:**

A total of 113 patients were included, and cefoperazone-induced coagulopathy occurred in 39 patients, with an incidence of 34.5%. These patients experienced significant prothrombin time prolongation around day 6 (median) after cefoperazone application. The serum trough concentration threshold of cefoperazone-induced coagulopathy in critically ill patients was 87.765 mg/l. Multivariate logistic regression analysis revealed that the APACHE II score (*p* = 0.034), prophylactic use of vitamin K_1_ (*p* < 0.001), hepatic impairment (*p* = 0.014), and C_min_ ≥ 87.765 mg/l (*p* = 0.005) were associated with cefoperazone-induced coagulopathy.

**Conclusion:**

Cefoperazone-induced coagulopathy usually occurs on the 6th day of cefoperazone use in critically ill patients. The risk will increase in patients with an APACHE II score > 25, hepatic impairment, and cefoperazone C_min_ ≥ 87.765 mg/l. Vitamin K_1_ is effective in preventing this adverse reaction.

## Introduction

Cefoperazone (CPZ) is a third-generation cephalosporin antibiotic that inhibits cell wall peptidoglycan synthesis by blocking the synthesis of peptidoglycan. This results in bacterial cell wall deficiency, leading to bacterial cell expansion and lysis. CPZ has a broad antibacterial spectrum with antibacterial activity against both Gram-positive and negative bacteria. However, the emergence of extended-spectrum β-lactamases (ESBLs) has limited its clinical application. Sulbactam (SAM) is an irreversible competitive β-lactamase inhibitor that protects β-lactam antibiotics from hydrolytic destruction. It has a bactericidal effect on *gonococci* and *Fusobacterium* spp. when used alone [1]. Cefoperazone sodium and sulbactam sodium for injection (CPZ/SAM) is a combination formulation that was introduced to the Chinese market in 1997. The combination of the two drugs had a significant synergistic effect, with a fourfold higher antibacterial effect than that of using CPZ alone [2]. It can be used to treat infections of the respiratory, digestive, and urinary tracts caused by common pathogens such as *Escherichia coli*, *Klebsiella pneumoniae*, *Pseudomonas aeruginosa*, *Acinetobacter baumannii*, and *Stenotrophomonas maltophilia* [3]. Due to its significant therapeutic effect and high safety profile, CPZ/SAM has been recommended as the first choice empirical treatment for various infectious diseases in China. However, with the widespread use of CPZ/SAM, the incidence of adverse reactions has increased. Since the 1980s, adverse events related to coagulation dysfunction caused by CPZ have been reported several times, and case reports of blood in urine, abdominal wall hematoma [4], and upper gastrointestinal bleeding [5] caused by CPZ have received widespread attention. Until 2019, NMPA has added the content of adverse reactions such as “coagulation disorders, bleeding” to the instructions of drugs containing CPZ.

Nevertheless, the threshold of CPZ exposure toxicity, especially the upper limit of serum concentration for CPZ-induced coagulation disorders, is unknown. Therefore, this study aimed to determine the serum concentration thresholds and risk factors for CPZ-induced coagulopathy in critically ill patients to reduce the incidence of bleeding events and mortality and to ensure patient safety with the drug.

## Materials and methods

### Study design

This was a single-center, retrospective, case-control study of critically ill patients from May 2021 and May 2023 at the intensive care unit of Drum Tower Hospital affiliated with the Medical School of Nanjing University [6]. All patients received CPZ/SAM (Sulperazon; Pfizer Inc, Shanghai, China, CPZ: SAM = 2:1) at a dosage of 3 g every 8 or 12 h. The dosage and prophylactic use of vitamin K_1_ were determined by the physician based on the patient’s pathophysiological condition. The inclusion criteria included patients ≥ 18 years of age and use of CPZ/SAM > 5 doses with monitoring of CPZ serum trough concentrations and monitoring of coagulation both before and after administration of the drug. Pregnant and lactating women, patients on anticoagulation therapy with warfarin, and patients with incomplete clinical records or relevant test data were excluded from the study.

### Data collection and definitions

Demographic and clinical data of patients meet the inclusion criteria through the hospital information system, including age, gender, diagnosis, acute physiology and chronic health evaluation (APACHE II), sequential organ failure assessment (SOFA), Nutric score, feeding status (general diet, liquid or semi-fluid, fasting), comorbidities (hypertension, diabetes, malignant tumors, cerebrovascular disease, hepatic impairment, renal disease, hematologic disease, autoimmune disease), surgery, continuous renal replacement therapy (CRRT), site of infection, bacterial type, dosage and duration of treatment, and serum trough concentration (C_min_) of CPZ. If the C_min_ is monitored several times during treatment, the maximum value will be taken. Laboratory indicators before CPZ medication including infection indicators, coagulation, liver function, renal function indicators, and other drugs used in combination that affect coagulation were obtained. Remedial measures should be taken according to the patient’s bleeding and coagulation function test results, including discontinuing medication and supplementing with vitamin K_1_, plasma, or other coagulation factors (cold precipitation, prothrombin complex). The coagulation function should be rechecked until it returns to normal.

An increased prothrombin time (PT) of 25% from baseline is defined as coagulopathy. Bleeding events are defined as having a bleeding-related diagnosis at discharge, but not at admission. Patients with adverse events were scored using the Naranjo scoring system to analyze the association between CPZ and coagulopathy. This study defines at least “possible” as CPZ-induced coagulopathy. Each case involving a suspected adverse event was independently reviewed by two researchers (one clinical pharmacist and one physician) for analysis.

### Measurement of CPZ serum concentration

After receiving at least 5 doses of CPZ therapy, the patient’s blood sample of 3 ml was collected before the next administration as C_min_. The sample was placed in an inert separation gel procoagulant vacuum blood collection tube, shaken, and centrifuged at 3000 r/min for 10 min, and the upper clear liquid was frozen and stored at −20 °C until analysis. If the patient adjusts the dosage, then the above method should be used to collect blood samples for measuring the C_min_. The determination of C_min_ is performed by validated liquid chromatography-tandem mass spectrometry (UPLC I-Class/Xevo TQD IVD system, Waters, USA), with an Acquity UPLC^®^BEH C18 1.7 μm IVD column (2.1 mm × 50 mm) as the chromatographic column. The mobile phase consists of (a) aqueous solution of formic acid and (b) methanol solution of formic acid, with gradient elution at a flow rate of 0.5 ml/min and a column temperature of 45 °C. The gradient program was as follows: 0–0.6 min, 95% A; 0.6–1.4 min, 95–20% A; 1.4–1.7 min, 20–5% A; 1.7–2.0 min, 5–95% A. Mass spectrometry is performed using an electrospray ionization source. The linear range of CPZ concentration was 0.2–20 mg/l, and the lower limit of quantification (LLOQ) is 0.02 mg/l, with precision and accuracy both within 15%.

### Statistical analyses

IBM SPSS Statistics, version 26.0 (IBM Corp., Armonk, N.Y., USA) and GraphPad Prism, version 8.0 (GraphPad Software, San Diego, California USA) were applied for analysis and graphing. For continuous variables, conformity to a normal distribution was expressed as mean ± standard deviation ($\overline{X }\pm S$), and continuous non-normally distributed measures were expressed as median (M) and interquartile range (IQR). Data were compared between two groups using either the *t* test (normal distribution) or the Mann–Whitney *U* test (non-normal distribution). Categorical variables were expressed as number (%), and comparisons between two groups were made using the Pearson chi-square test, continuity correction test, or Fisher exact test. Factors with *p* < 0.1 in the baseline variables analysis were included in the forward multivariate logistic regression analysis, and odds ratios (OR) and 95% confidence intervals (CIs) were calculated. A *p*-value of < 0.05 indicated statistical significance. The receiver operating characteristic (ROC) curve was used to determine the threshold of C_min_ of CPZ that causes coagulation disorders in critically ill patients.

## Results

### Demographics and clinical characteristics

**Fig. 1:**
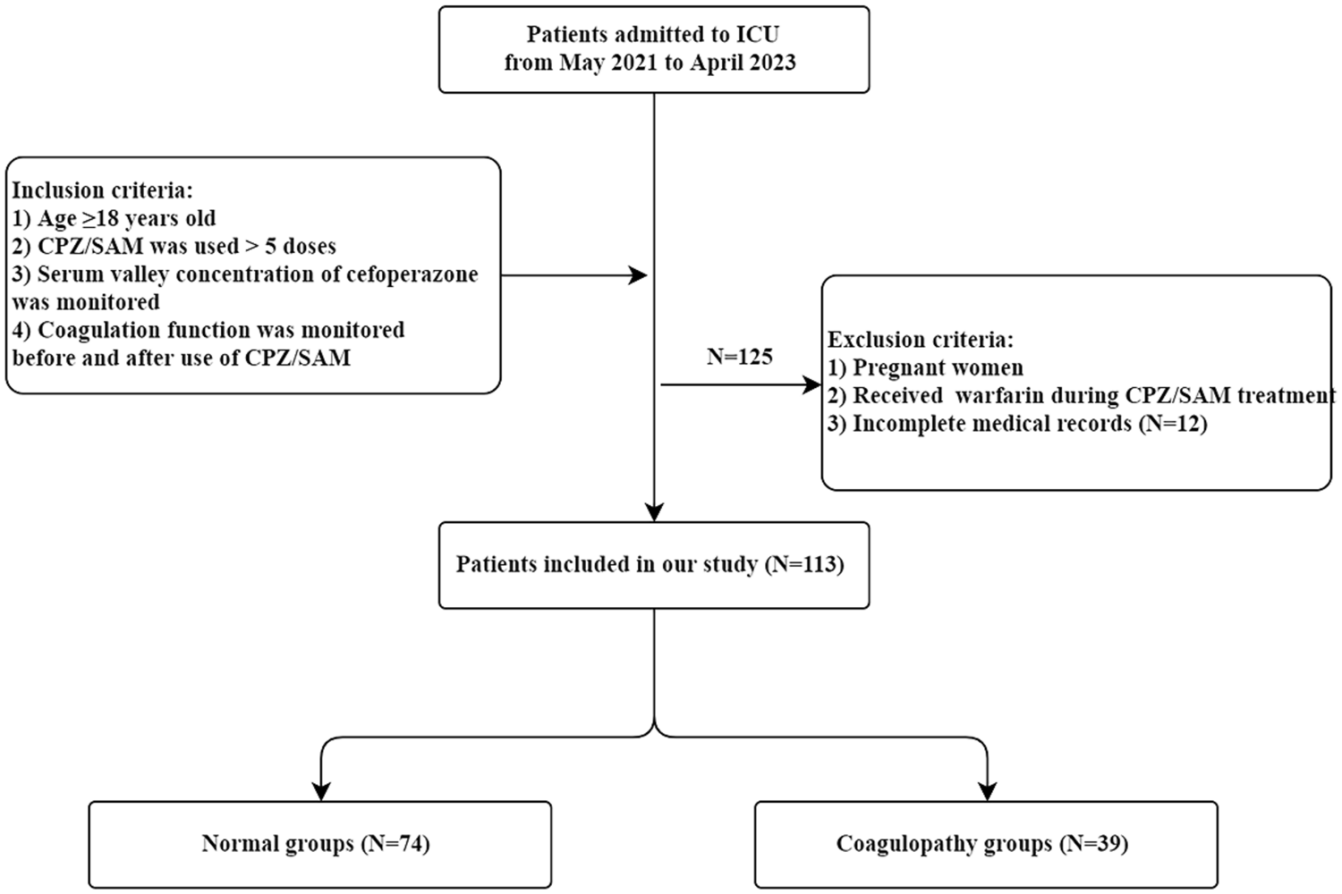
Flowchart of patients included in this study. ICU: intensive care unit; CPZ/SAM: cefoperazone sodium and sulbactam sodium

From May 2021 to April 2023, a total of 125 critically ill patients in our ICU met the inclusion criteria. Among them, 12 patients were excluded based on exclusion criteria, including 8 patients who received less than 5 doses of CPZ/SAM, 3 patients who did not measure C_min_, and 1 patient who did not have coagulation indicators monitored after medication. Ultimately, a retrospective analysis was conducted on 113 patients who were included (Fig. 1). The predominant population of patients was male (67.3%), with an average age of 68.05 ± 17.23 years. In addition to empirical medication (45.1%), the primary site of infection was the lungs (35.4%). In infections confirmed by microbiology, the primary pathogen was *Acinetobacter baumannii* (25.7%), and patients infected with two or more pathogens account for 14.2%. A total of 39 patients (coagulopathy group) developed coagulation disorders after receiving treatment with CPZ/SAM, while 74 patients did not develop coagulation disorders (normal group), resulting in a coagulation disorder incidence rate of 34.5%. The baseline characteristics of the two groups of patients are shown in Table 1, including gender, age, SOFA score, infection site, pathogenic microorganisms, dosage, treatment duration, and eating status, which were not statistically different (*p* > 0.05).

**Table 1:** Bivariate comparisons of clinical characteristics between normal groups and coagulopathy groups

Variables	Normal groups (*n* = 74)	Coagulopathy groups (*n* = 39)	${x}^{2}$/*t*/*Z*	*p*
Sex, male, *n* (%)	46 (62.2)	30 (76.9)	2.527	0.112
Age, years, mean ± SD	69.49 ± 16.11	65.33 ± 19.11	1.158	0.251
APACHE II score	23 (19–27.25)	27 (21–31)	−1.860	**0.063**
SOFA score	8.61 ± 4.52	9.92 ± 4.68	−1.452	0.149
Surgery (%)	27 (36.5)	9 (23.1)	2.115	0.146
Duration of CPZ therapy (days)	10.5 (7–16)	10 (7–15)	−0.230	0.818
Dose (2 g q8/12 h)	52 / 22	23 / 16	1.460	0.227
Prophylactic use of vitamin K_1_ (%)	37 (50.0)	5 (12.8)	15.117	**0.000**
CRRT (%)	21 (28.4)	14 (35.9)	0.675	0.411
Comorbidities (%)				
Diabetes	15 (20.3)	10 (25.6)	0.428	0.513
Hypertension	31 (41.9)	22 (56.4)	2.162	0.141
Malignant tumors	5 (6.8)	4 (10.3)	0.083	0.773
Hepatic impairment	6 (8.1)	14 (35.9)	13.540	**0.000**
Renal disease	17 (23)	13 (33.3)	1.406	0.325
Hematologic disease	3 (4.1)	-	0.410	0.236
Immune system disease	3 (4.1)	1 (2.6)	0.000	1.000
Shock	10 (13.5)	8 (20.5)	0.934	0.334
Infection site (%)				
Pneumonia infection	25 (33.8)	15 (38.5)	0.244	0.621
Bloodstream infection	2 (2.7)	1 (2.6)	0.000	1.000
Urinary tract infection	1 (1.4)	1 (2.6)	0.000	1.000
Intra-abdominal infection	2 (2.7)	1 (2.6)	0.000	1.000
Empirical medication	36 (48.6)	15 (38.5)	1.070	0.301
Others	2 (2.7)	1 (2.6)	0.000	1.000
Two or more than	5 (6.8)	4 (10.3)	0.083	0.773
Pathogenic microorganism (%)				
*Escherichia coli*	1 (1.4)	1 (2.6)	0.000	1.000
*Pseudomonas aeruginosa*	5 (6.8)	3 (7.7)	0.000	1.000
*Acinetobacter baumannii*	17 (23)	12 (30.8)	0.814	0.367
*Klebsiella pneumoniae*	1 (1.4)	3 (7.7)	1.437	0.231
Empirical medication	36 (48.6)	16 (41)	0.597	0.440
Two or more than	12 (16.2)	4 (10.3)	0.746	0.388
Feeding status (%)				
General diets	14 (18.9)	11 (28.2)	1.278	0.258
Liquid or semi-fluid	36 (48.6)	21 (53.8)	0.276	0.599
Fasting	24 (32.4)	7 (17.9)	2.691	0.101
Nutric score	6 (4–7)	7 (5–8)	−1.943	**0.052**
Combination therapy (%)				
Heparin/low molecular heparin	44 (59.5)	17 (43.6)	2.589	0.108
NSAIDs	6 (8.1)	3 (7.7)	0.000	1.000
Glucocorticoids	26 (35.1)	14 (35.9)	0.006	0.936
Antiplatelet agents	8 (10.8)	5 (12.8)	0.000	0.993
Tigecycline	8 (10.8)	10 (25.6)	4.194	**0.041**
Vancomycin	8 (10.8)	8 (20.5)	1.978	0.116
Moxifloxacin	5 (6.8)	2 (5.1)	0.000	1.000
Linezolid	17 (23)	5 (12.8)	1.679	0.195
SMX	4 (5.4)	2 (5.1)	0.000	1.000
Voriconazole	11 (14.9)	10 (25.6)	1.960	0.161
Caspofungin	11 (14.9)	3 (7.7)	0.610	0.424
Immunosuppressant	2 (2.7)	2 (5.1)	0.016	0.898
Sodium valproate	8 (10.8)	3 (7.7)	0.039	0.843
Gabexate mesylate	-	3 (7.7)	3.250	**0.071**
WBC (× 10^9^/l)	11.4 (8.18–14.75)	12.2 (7.6–15.1)	−0.069	0.945
Neu%	84.3 (73.15–92.7)	87.7 (79.8–91.9)	−0.667	0.505
Hb (g/l)	92 (79.75–111.25)	93 (77–104)	−0.341	0.733
PCT (ng/ml)	0.75 (0.16–2.293)	1.009 (0.466–4.955)	−1.536	0.125
IL-6 (pg/ml)	51.97 (23.73–143.61)	103.38 (45.06–218.47)	−2.214	**0.027**
CRP (mg/l)	51.7 (28.4–94.25)	80.1 (35.5–136.1)	−1.695	**0.090**
PT (s)	13 (11.78–14.15)	12.6 (11.7–14.5)	−0.468	0.690
AST (U/l)	33 (20.2–50.3)	34.9 (20.3–59)	−0.308	0.758
TB (µmol/l)	11.8 (7.9–21.55)	11.7 (7–25)	−0.085	0.933
ALB (g/l)	32.6 (30.9–35.525)	32.9 (29.6–38.6)	−0.154	0.878
CLcr (ml/min)	58.5 (23.1–102)	33.0 (20.55–63.55)	−1.679	**0.093**
HLA-DR (%)	39.0 (24.53–63.53)	40.5 (28.5–53.95)	−0.219	0.826
C_min_ (mg/l)	73.085 (35.75–133.73)	137.6 (73.30–180.80)	−2.950	**0.003**

Variables with *p* < 0.1 are indicated in boldface

*APACHE II* Acute Physiology and Chronic Health Evaluation II, *ALT* alanine aminotransferase, *AST* aspartate aminotransferase, *C*_*min*_ serum concentration of 30 min before next dose, *CLcr* creatinine clearance, *CRP* c-reactive protein, *Hb* hemoglobin, *HLA-DR* human leukocyte antigen DR, *IL-6* Interleukin-6, *NSAIDs* non-steroidal anti-inflammatory drugs, *Neu%* neutrophil percentage, *PCT* procalcitonin, *PT* prothrombin time, *SOFA* sequential organ failure assessment, *SMX* compound sulfamethoxazole, *TB* total bilirubin, *WBC* white blood cell count

### Effect of CPZ on coagulation and clinical outcomes

**Fig. 2:**
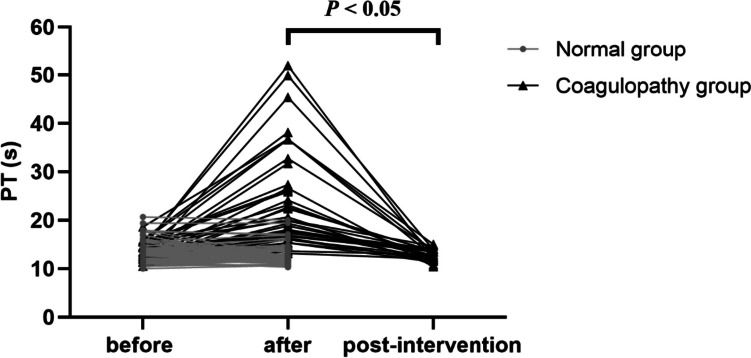
Comparison of pre-treatment, post-treatment, and post-intervention PT values for cefoperazone

**Fig. 3:**
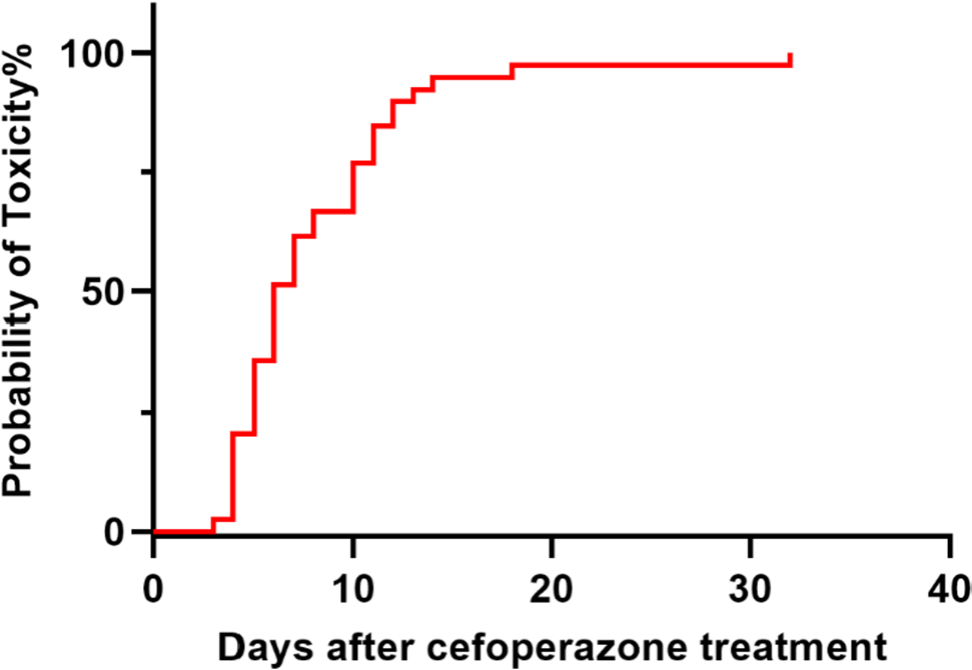
Kaplan-Meier plot showing the time from the initiation of cefoperazone therapy to the development of coagulopathy

Two groups of patients’ coagulation indexes before and after CPZ treatment are shown in Table 2. There was no significant difference in PT between the two groups at baseline (*p* = 0.690). However, after CPZ treatment, the PT of patients with coagulopathy was significantly prolonged to 19.9 (16.7–27.3) seconds compared to 12.6 (11.7–14.5) seconds before medication (*p* = 0.042). After administering vitamin K_1_ or other blood products, the PT level was significantly reduced (*p* < 0.05), as shown in Fig. 2. The Kaplan-Meier curve shows that the median time from the start of treatment to the induction of coagulation disorders by CPZ is 6 days (Fig. 3). As the duration of use increased, the number of patients with abnormal coagulation function gradually increased. After 14 days of CPZ treatment, 32.74% of patients experienced coagulation disorders.

**Table 2:** Coagulation index of patients before and after cefoperazone administration

Variables	Normal groups			Coagulopathy groups		
	Before	After	*p*	Before	After	*p*
PT	13 (11.78–14.15)	12.45 (11.6–13.83)	0.042	12.6 (11.7–14.5)	19.9 (16.7–27.3)	0.000
APTT	30.2 (28.35–35.03)	29.45 (27.05–34.23)	0.006	30.4 (27.8–36)	43 (35.3–54.7)	0.000
INR	1.15 (1.04–1.26)	1.1 (1.02–1.18)	0.006	1.13 (1.03–1.31)	1.71 (1.46–2.41)	0.000
Fib	3.55 (2.3–5.15)	4.1 (2.98–5.4)	0.079	4.4 (2.7–5.5)	3.0 (1.9–4.8)	0.060
PLT	163 (97–298.25)	169 (75.25–232.75)	0.086	181 (70–247)	94 (52–153)	0.000
D-D	3.12 (1.50–7.15)	2.60 (1.34–5.12)	0.010	3.45 (1.75–7.7)	2.88 (1.73–6.64)	0.068

*APTT* activated partial thromboplastin time, *D-D* D-dimer, *Fib* fibrinogen, *INR* international normalized ratio, *PLT* platelet, *PT* prothrombin time

Among patients with coagulation dysfunction, a total of 8 patients experienced bleeding of varying degrees, including 5 with gastrointestinal bleeding, 1 with diffuse alveolar hemorrhage, and 1 with local bleeding of the right iliac muscle. The incidence of bleeding was significantly different compared to the normal group (*p* = 0.001). In terms of blood product infusion, patients with coagulopathy took corresponding remedial measures after experiencing adverse reactions, and the infusion frequency was significantly higher than that of the normal group (*p* < 0.001). Compared with the normal group, the ICU hospitalization time (*p* = 0.454), recovering rate (*p* = 0.301), and automatic discharge rate (*p* = 0.243) of the coagulopathy group were not statistically significant, but the 28-day all-cause mortality was significantly higher than that of the normal group (*p* = 0.01) (Table 3).

**Table 3:** Hemorrhagic events and outcomes of patients treated with cefoperazone in ICU

Variables	Normal groups	Coagulopathy groups	${x}^{2}$/*Z*	*p*
Bleeding (%)	1 (1.4)	8 (20.8)	10.312	0.001
Transfusion blood products (%)	2 (2.7)	11 (28.2)	13.906	0.000
ICU hospitalization time (days)	25 (14.75–42.25)	29 (13–55)	−0.749	0.454
Recovering (%)	36 (48.6)	15 (38.5)	1.070	0.301
Automatic discharge (%)	27 (36.5)	10 (25.6)	1.364	0.243
28-day all-cause mortality (%)	11 (14.9)	14 (35.9)	6.557	0.010

### Risk factors for CPZ-associated coagulopathy

**Fig. 4:**
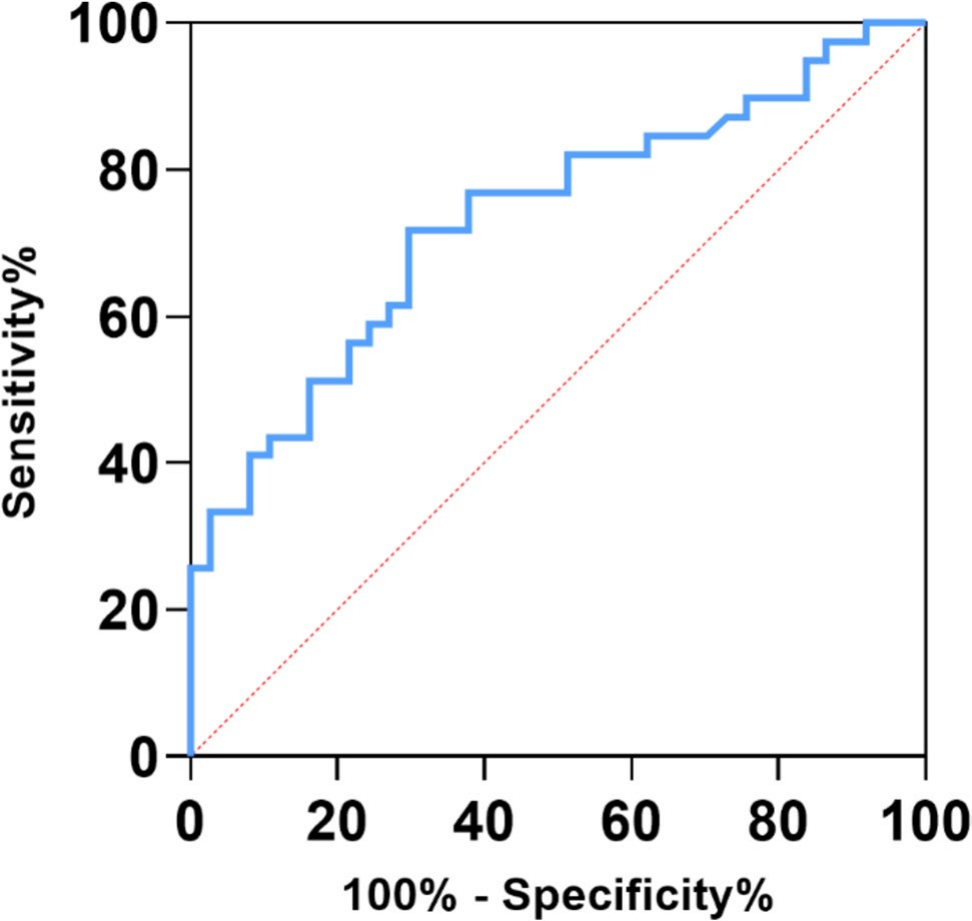
Receiver operating characteristic curve analysis of cefoperazone-associated coagulopathy concentration thresholds

In the analysis of baseline data of patients, the development of coagulopathy caused by CPZ was related to APACHE II score (*p* = 0.063), vitamin K_1_ prevention (*p* < 0.001), concurrent liver dysfunction (*p* < 0.001), Nutric score (*p* = 0.052), combined use of tigecycline (*p* = 0.041), gabapentin mesylate (*p* = 0.071), IL-6 level (*p* = 0.027), CRP level (p = 0.09), and creatinine clearance rate (CLcr) (*p* = 0.093). In the study, the C_min_ of the normal group and the coagulopathy group were found to be 73.085 (35.75–133.73) mg/l and 137.6 (73.3–180.8) mg/l, respectively. The difference between the two groups was statistically significant (*p* = 0.003). Furthermore, based on the ROC curve (Fig. 4), the C_min_ cut-off value associated with an increased risk of coagulation disorders was found to be 87.765 mg/l. The area under the ROC curve (AUC) was calculated to be 0.734, with a sensitivity of 71.8% and a specificity of 70.3%. Variables with a *p*-value < 0.1 were included in a multiple forward logistic regression analysis (Table 4). The results indicated that upon admission to the ICU, the APACHE II score (OR, 3.229; *p* = 0.034), prophylactic use of vitamin K_1_ (OR, 0.08; *p* < 0.001), co-existing hepatic impairment (OR, 5.616; *p* = 0.014), and C_min_ ≥ 87.765 mg/l (OR, 5.045; *p* = 0.005) are independent factors affecting the occurrence of CPZ-induced coagulopathy in patients.

**Table 4:** Multivariate analysis of risk factors for cefoperazone-associated coagulopathy

Variables	β	SE	Wald	OR	95%CI	*p*
APACHE II > 25	1.172	0.554	4.471	3.229	1.089–9.57	0.034
Prophylactic use of vitamin K_1_	−2.532	0.706	12.876	0.08	0.02–0.317	0.000
Hepatic impairment	1.726	0.706	5.98	5.616	1.409–22.392	0.014
C_min_ ≥ 87.765 mg/l	1.618	0.58	7.791	5.045	1.619–15.716	0.005

*OR* odds ratio, *CI* confidence interval

## Discussion

Coagulopathy is a more serious adverse reaction during the use of CPZ-containing drugs, and the incidence of coagulation dysfunction in patients using CPZ/SAM has been reported to be about 25.8% or even up to 45% in clinical practice [7]. The main manifestations are reduced prothrombin activity, prolonged prothrombin time, and thrombocytopenia [8, 9]. Grasela et al. [10] concluded that patients with severe disease are at the highest risk of developing coagulopathy. Due to their specific pathophysiological situation, critically ill patients may already be in a relatively disturbed state of coagulation. This phenomenon is mainly associated with endothelial cell damage caused by factors such as the release of inflammatory transmitters and activation of leukocyte adhesion factors in patients, or with the inflammatory response of patients [11]. At the same time, the pharmacokinetics of CPZ in critically ill patients undergo significant changes [12], which increases the risk of CPZ-induced coagulation dysfunction. Patients with severe coagulopathy not only experience a significant increase in bleeding events and transfusion volume but are also more likely to develop multiple organ dysfunction syndrome, with a mortality rate more than 4 times higher than that of patients with normal coagulation function [13]. This study showed that the incidence of coagulation dysfunction induced by CPZ in critically ill patients is 34.5%, with a bleeding event rate of 7.08%. Patients in the coagulopathy group experienced significant PT prolongation around 6 days (median) after CPZ application.

This study attempted to determine the threshold of toxic exposure to CPZ by therapeutic drug monitoring (TDM). To our knowledge, this is the first study on the concentration-response relationship of CPZ-induced coagulation disorders. Previous studies only found that CPZ was associated with an increased risk of hypoprothrombinemia and bleeding, and there was a dose–response relationship. Chen et al. [14] suggested that patients who used anticoagulants, suffered from liver failure, or malnutrition, and had a history of bleeding events faced a significantly increased risk of bleeding events. They calculated the cumulative dose of CPZ based on a defined daily dose (DDD), and when the cumulative dose exceeded 5 DDDs, the incidence of bleeding events was even higher. In a retrospective cohort study involving 476 patients, the risk of prolongation of PT was found to increase fivefold in the high-dose group of CPZ (> 4.5 g/d) compared to the cefotaxime or ceftizoxime group and 16-fold compared to the ceftazidime group [15]. Andrassy et al. [16] also demonstrated that in the presence of high CPZ serum C_min_, a platelet defect with prolonged bleeding time and impaired platelet aggregation may occur. In this study, the C_min_ threshold for CPZ-induced coagulation dysfunction was 87.765 mg/l, and the AUC was 0.734, which had a good predictive value by plotting the ROC curve. Moreover, the results of multivariate logistic regression analysis showed that cefepime C_min_ ≥ 87.765 mg/l was associated with a fivefold increase in the risk of CPZ-induced coagulopathy in patients.

Vitamin K is an essential cofactor for the carboxylation of hepatic cell mitochondria. It is involved in the γ-carboxylation reaction of glutamic acid in prothrombin precursors and is mainly obtained from daily dietary intake [17]. Many studies have suggested that there are two main mechanisms for CPZ-induced coagulopathy: (1) CPZ is hardly metabolized in the body, with about 75% excreted in bile, inhibiting the growth of normal intestinal flora and reducing the synthesis of vitamin K in the intestines; (2) the N-methylthiotetrazole (NMTT) side chain carried by CPZ has a structure similar to glutamic acid, which can interfere with the carboxylation of vitamin K in the liver, thereby reducing the synthesis of prothrombin and lowering the levels of coagulation factors II, VII, IX, and X that depend on vitamin K [18, 19]. Nevertheless, prophylactic use of vitamin K_1_ prior to CPZ therapy remains controversial. In a retrospective cohort study of 374 patients treated with CPZ in a teaching hospital, prophylactic use of vitamin K did not reduce bleeding [15]. Rockoff et al. [20] concluded that routine use of vitamin K and CPZ for perioperative infection prophylaxis may not be necessary. The results of a clinical trial showed that 141 patients who received CPZ without prophylactic vitamin K did not bleed [21]. The logistic regression results of this study indicated that the prophylactic application of vitamin K_1_ is an independent protective factor for preventing CPZ-induced coagulation disorders. Prophylactic administration of vitamin K_1_ can significantly prevent patients’ coagulation disorders, even when exposed to higher concentrations of CPZ.

Currently, there were many studies on the factors influencing the abnormal coagulation function caused by CPZ, and a large number of studies believed that the risk of this coagulopathy is closely related to the patient’s own pathological and physiological conditions. Advanced age, cancer, malnutrition, underlying hepatic impairment, renal insufficiency, combined use of anticoagulants, and previous bleeding history were all considered risk factors for CPZ-induced coagulopathy [7, 22]. The results of this study showed that combined hepatic impairment was an independent risk factor for coagulation dysfunction in patients (OR, 5.616; *p* = 0.014), which was consistent with previous studies. Hepatic insufficiency can lead to reduced drug metabolism and accumulation in the body, as well as a decreased ability of the liver to synthesize its own coagulation factors, leading to abnormal coagulation [22]. In a study of 35 patients with severe infections and renal dysfunction [23], in non-jaundiced patients with abnormal liver function, the mean peak and trough serum concentrations produced by a 2-g dose every 12 h were 254 and 125 mg/l, respectively, while in five tests with normal liver function, they were 179.5 and 19.5 mg/l, respectively. Although the trough value increased significantly, there was no statistical significance (*p* > 0.1).

In this study, an APACHE II score > 25 was also found to be associated with CPZ-induced coagulopathy (OR, 3.229; *p* = 0.034). The APACHE II score is currently the most widely used system for assessing the condition and prognosis of critically ill patients in clinical settings and can serve as an indicator for evaluating the condition and prognosis of patients in intensive care units [24]. It has been shown that critically ill patients with an APACHE II score of > 10 are at high risk for severe malnutrition [25]. This also explained why patients with higher APACHE II scores are more likely to experience CPZ-related coagulation dysfunction. However, when the Nutric score (*p* = 0.052) was included in a multivariate logistic regression, it was progressively eliminated from the model. This may be related to the bias in the definition of malnutrition and nutrition scores of the included population. Meanwhile, there was no statistical difference in gender between the two groups (*p* = 0.052). However, in previous studies, results showed that when including patients under 12 years old, the incidence of antibiotic-associated hypoprothrombinemia in males was higher than in females. They concluded that according to the effect of sex hormones on prothrombin, in the presence of estrogen, prothrombin formation is faster, and the effective concentration of vitamin K is lower. The level of prothrombin is higher in women than in men, and the dietary requirement of vitamin K is lower in women [26]. Perhaps because of differences in the included populations, this study could not reach similar conclusions. Cohort studies with larger sample sizes may be needed regarding the effect of sex hormones on prothrombin.

Drugs that may cause coagulation disorders in patients were also included in the analysis, including antiplatelet agents, other antimicrobials, drugs that may cause bleeding (NSAIDs and glucocorticoids), and anticoagulants (heparin and new oral anticoagulants) [27]. Univariate analysis showed that the combined use of tigecycline was associated with prolonged PT in patients. According to current researches, tigecycline could cause prolongation of PT and APTT, elevation of INR, decreased platelet count [28], and hypofibrinogenemia. Hu et al. [29] analyzed the characteristics of patients with hypofibrinogenemia who received treatment with CPZ/SAM and found that these patients had a higher incidence of coagulation abnormalities (*p* = 0.009) and required more blood products (*p* = 0.003). However, this study did not obtain similar results, possibly due to the small sample size and the use of vitamin K_1_ for prevention, indicating the need for large-scale cohort studies to draw conclusions. Several recent studies have also yielded similar conclusions [30–32]. Furthermore, Miao et al. [31] also investigated the effect of the combined use of sodium valproate on CPZ-induced coagulation disorders, and the results showed that sodium valproate is not associated with this adverse reaction, which is consistent with our research findings. Low-dose aspirin is commonly used for antiplatelet therapy, but its antiplatelet mechanism may lead to an increased risk of bleeding. When patients on long-term aspirin use CPZ for antibiotic therapy, the combination of CPZ and aspirin can have a cumulative effect on coagulation dysfunction, resulting in a significant increase in the risk of bleeding [33]. However, this study has not yielded similar conclusions, possibly because the duration of combined use of aspirin and CPZ in ICU patients was insufficient for us to observe the occurrence of this phenomenon. A prospective observational study has also concluded that the combination of vancomycin, a commonly used drug for Gram-positive bacteria in the ICU, with CPZ could lead to a significantly higher risk of coagulation disorders [34]. Although this study did not find any drug combination as a risk factor for CPZ-induced coagulation dysfunction, caution should still be exercised in clinical practice regarding the potential bleeding risk associated with these drugs.

Obviously, the patient’s own risk factors are an important component of adverse reactions, and higher serum C_min_ levels of CPZ amplify these inducing factors [35]. This study has certain limitations. Firstly, this is a single-center, retrospective case-control study, which cannot exclude the bias of medical monitoring. Secondly, the heterogeneity of critically ill patients limited our statistical analysis. The serum C_min_ threshold of coagulation disorders caused by CPZ needs further multicenter, prospective, randomized controlled studies to determine. Thirdly, this is a study aimed at the Chinese population and does not involve discussion of other races. We did not conduct genetic testing on the patients, and in fact, pharmacogenetics is also an area worth exploring. For example, since CPZ is mainly excreted via bile, it has been suggested that multidrug resistance-associated protein (MRP) 2 (ABCC2) is the transporter protein primarily responsible for CPZ excretion. In patients with hereditary MRP2 (ABCC2) expression deficiency, if there is no compensatory efflux mediated by other transporter proteins, it may lead to obstruction of CPZ excretion in bile, resulting in elevated blood drug concentration and potential occurrence of coagulopathy [36, 37]. Not only that but due to the possibility of vitamin K epoxide reductase (VKOR) being one of the targets of NMTT [18], we are unable to rule out its pharmacogenetic effects. Whether single nucleotide polymorphisms (SNPs) in VKOR increase CPZ sensitivity and lead to coagulation disorders in patients, further genomic research may be needed to confirm this hypothesis. Nevertheless, our study results still provide valuable information about the serum C_min_ threshold of coagulopathy caused by CPZ in real-world patients.

In conclusion, we found that the incidence of coagulopathy in critically ill patients treated with CPZ was 34.5%, with a higher likelihood of bleeding events. The median time for the occurrence of coagulation dysfunction was 6 days, and the CPZ serum C_min_ threshold was 87.765 mg/l. Prophylactic application of vitamin K_1_ significantly reduces the incidence of this adverse reaction in critically ill patients. We recommend that critically ill patients with an APACHE II score > 25 and combined hepatic impairment should have timely TDM and coagulation monitoring with the application of CPZ-containing drugs, and it would be more prudent to apply vitamin K1 prophylaxis, while ensuring the efficacy of CPZ treatment in order to prevent coagulopathy and fatal bleeding events.

## Data Availability

The data used and/or analyzed in this study are available from the corresponding author on reasonable request.
